# The Ability of an Algoclay-Based Mycotoxin Decontaminant to Decrease the Serum Levels of Zearalenone and Its Metabolites in Lactating Sows

**DOI:** 10.3389/fvets.2021.704796

**Published:** 2021-09-30

**Authors:** Xandra Benthem de Grave, Janine Saltzmann, Julia Laurain, Maria A. Rodriguez, Francesc Molist, Sven Dänicke, Regiane R. Santos

**Affiliations:** ^1^Schothorst Feed Research, Lelystad, Netherlands; ^2^Institute of Animal Nutrition, Friedrich-Loeffler-Institute (FLI), Federal Research Institute for Animal Health, Brunswick, Germany; ^3^Olmix SA, Brèhan, France

**Keywords:** milk, colostrum, biological samples, pig, fusarium, mycotoxins

## Abstract

This study evaluated the effect of an algoclay-based mycotoxin decontaminant on the levels of ZEN, DON, and their derivatives in the colostrum, milk, and serum of sows, as well as in the serum of weaned piglets after maternal mycotoxin exposure during the last week of gestation and during lactation of sows (26 days). For this, sows (*n* = 5) were fed diets artificially contaminated with 100 (LoZEN) or 300 (HiZEN) ppb ZEN, with or without an algoclay-based mycotoxin decontaminant in the highly contaminated diet. All diets contained 250 ppb deoxynivalenol (DON). Dietary treatments did not affect the performance of the sows and piglets. Only α-ZEL was significantly increased in the colostrum of sows fed the HiZEN diet, and this increase was even higher in the colostrum of the sows fed the HiZEN diet supplemented with the test decontaminant. However, no differences in milk mycotoxin levels were observed at weaning. The highest levels of ZEN, α-ZEL, and β-ZEL were observed in the serum of sows fed the HiZEN diet. When the HiZEN diet was supplemented with the tested algoclay-based mycotoxin decontaminant the levels of ZEN and its metabolites were significantly decreased in the serum of sows. Although all sows were fed the same levels of DON, the serum level of de-epoxy-DON was increased only in the serum of piglets from the sows fed a diet with the non-supplemented HiZEN diet. In conclusion, the tested algoclay-based mycotoxin decontaminant can decrease the levels of ZEN and its metabolites in the serum of sows and the level of de-DON in the serum of piglets.

## Introduction

Mycotoxins are toxic compounds produced by different fungal species, *Fusarium* being responsible for most contamination of feedstuffs in the field. Among more than 400 identified mycotoxins, deoxynivalenol (DON) and zearalenone (ZEN) are of most concern for the pig industry. Pigs are very sensitive to both DON and ZEN. When acute dietary exposure occurs, DON can lead to vomiting and feed refusal (1), whereas ZEN has oestrogenic effects (2). However, in practice, acute toxicity occurs occasionally, and chronic dietary exposure plays the main role in economic losses, especially when young piglets are exposed to these mycotoxins. Such exposure may even occur via the placenta and/or during lactation (3). The presence of DON and its metabolite de-epoxy-DON (de-DON) was detected in the colostrum of sows (4, 5). Recently, DON, ZEN, and their metabolites were identified in the colostrum, milk, and serum of sows fed diets containing 250 ppb DON and 100 or 300 ppb ZEN (6). In this earlier study, we also confirmed the transmission of these mycotoxins during the last week of gestation and lactation by measuring mycotoxin levels in the serum of suckling piglets. The gestation and lactation exposure did not impair the performance of the piglets but an inflammatory process was taking place.

To limit exposure, diets should be prepared with grains containing low levels of mycotoxins. However, feed is prepared with different feedstuffs that will present variable levels of different mycotoxins. Therefore, feed additives capable of inactivating these mycotoxins or preventing their absorption by the animals may help prevent outbreaks and financial losses. Bentonites are widely used for this purpose and registered in the EU for their capacity to bind aflatoxin B1 (7). Bentonites are phyllosilicates with variable physicochemical properties (8, 9) concluded there are significant correlations between *in vitro* ZEN adsorption and both the d-spacing (interlayer space) and mineral fraction at different pH levels. Another study indicated that increasing the bentonite interlayer space thanks to alkyl groups may improve the adsorption of mycotoxins (10). However, the use of alkyl groups in animal feed is prohibited in Europe. The polysaccharide ulvan, present in the cell wall of green seaweed (Chlorophyceae) of the genus *Ulva*, is a natural compound able to increase the bentonite interlayer space (11). An algoclay technology composed of seaweed extract and bentonite has therefore been developed (12).

The aim of the present study was to evaluate the efficacy of the algoclay-based mycotoxin decontaminant against the transmission of ZEN and DON from sows to piglets during the last week of gestation and lactation. For this, sows were fed diets naturally contaminated with ZEN (300 ppb) and DON (250 ppb).

## Materials and Methods

### Animals and Housing

This study was performed alongside another trial evaluating the effect of ZEN transmission from sows to piglets during lactation (6). To reduce the number of experimental animals, part of the control data from the present trial was shared with this former study. A total of 15 clinically healthy sows (Landrace x Large White; parity 1 to 6) with normal body condition were used in this experiment. Sows had an average parity of three and parity was balanced across treatments as much as possible. During gestation, sows were housed in groups of approximately 150 sows with four feeding stations available per group. Sows were fed a commercial diet (Supplementary Table 1) during gestation with marginal levels of mycotoxins. Sows were transferred from the gestation unit to the farrowing rooms at day 109 of gestation and fed the experimental diets (Supplementary Table 2). The farrowing pens (0.60 x 2.50 m for the sow; 2.25 x 2.50 m total area) were equipped with a feeding bin and the sows were able to fill the feeder by pushing a metal bar in the feeder. Sows did not have access to straw or other bedding material. Pens had a plastic slatted floor, including a heated section for piglets programmed to reach 40°C at farrowing, reducing to 30°C at 3 weeks after birth. The room temperature was scheduled to decrease from 24°C at farrowing to 20°C at 5 days after farrowing. Artificial light was provided from 6:00 to 22:00 hours. From day 14 after birth and until weaning, all the piglets received creep feed (Supplementary Table 3), which was not contaminated with mycotoxins.

### Diets and Experimental Design

The experimental diets were prepared with naturally contaminated feedstuffs and, as a consequence, the *Fusarium* mycotoxin DON was present in all of them at the same level (~250 ppb). Two batches of sugar beet pulp were used as a source of ZEN in the present study. One had negligible contamination, while the other was highly contaminated with ZEN. All other main feedstuffs (corn, soybean meal, wheat, sunflower seed meal, soybean hulls, and clean beet pulp batch) were present at the same inclusion levels in all diets. As a result, the experiment had two main ZEN levels in the diets (low: ~100 ppb or LoZEN; high: ~300 ppb or HiZEN). The HiZEN diet was divided into two sub-batches were one of them was supplemented with the tested mycotoxin decontaminant, which consisted of an algoclay technology using the water-soluble polysaccharide ulvan, present in the cell wall of green seaweed (Chlorophyceae) of the genus Ulva, (13) and montmorillonite (layer clay) was used in this study. This material was prepared following the patent EP2674397 with at least 60–90% montmorillonite and 40–10% seaweed on dry matter basis and providing an interlayer space up to 3 nm, manufactured by Olmix, S.A. The recommended maximum level of ZEN in sow diets is 250 ppb (14).

The experiment comprised three dietary treatments and five replicates per treatment, where each sow was a replicate. Treatments were randomly allocated to the sows. 1 week before farrowing, sows were moved to the lactation unit and the gestation diet was replaced by the respective experimental diets (Table 1). From day 14 after birth until weaning (day 26) all piglets received an uncontaminated commercial creep feed. Sows and piglets were monitored daily for abnormalities, such as abnormal behavior, clinical signs of illness, and mortality throughout the experiment.

**Table 1:** Experimental treatments.

**Treatments**	**Day 109 of gestation to 26 days of lactation**
1	LoZEN
2	HiZEN
3	HiZEN + 0.15% algoclay-based mycotoxin decontaminant

*All diets also contained ~250 ppb DON, regardless of the treatment. LoZEN: ~100 ppb; HiZEN: ~300 ppb*.

All diets were analyzed in an independent and accredited (BELAC 057-TEST/ISO17025) laboratory (Primoris Holding, Gent, Belgium) via liquid chromatography with tandem mass spectrometry (LC-MS/MS). This multi-mycotoxin analysis confirmed that ZEN was the main contaminant, followed by DON, while other mycotoxins were found at low to negligible levels. Importantly, although all diets also contained low levels of DON, this mycotoxin was at a constant background level in all three diets. Values of all detected mycotoxins in the diets are presented in Table 2.

**Table 2:** Multi-mycotoxin analyses of the diets (levels in ppb).

	**Diet**		
**Mycotoxin (ppb)**	**LoZEN**	**HiZEN**	**HiZEN + decontaminant**
	**(T1)**	**(T2)**	**(T3)**
ZEN	118	318	318
DON	259	255	245
Fumonisin B1 + B2	83.1	84.0	119
AOH	26.9	29.5	39.4
AME	68.5	68.6	69.3
Beauvericin	19.8	27.7	28.9
Enniatin A1	3.5	-	-
Enniatin B	32.6	28.9	31.5
Enniatin B1	9.2	8.6	9.6

*Below detection level in all diets, Aflatoxin B1, B2, G1, and G2, 3 + 15 Acetyl deoxynivaloenol (Ac-DON), Deoxynivalenol 3-Glucoside (DON-3-G), Nivalenol, Ochratoxin A, T2 & HT2 Toxin, Diacetoxyscirpenol, Cytochalasine E, Sterigmatocystin, Alternariol ME, Citrinin, Roquefortine C, Enniatin A, A1, B, and B1, Moniliformin. ZEN, zearalenone; DON, Deoxynivalenol; AOH, Alternariol; AME, Alternariol monomethyl ether*.

### Measurements

#### Performance

Sows were individually weighed at day 109 of gestation, on the day of farrowing, and at weaning. Backfat thickness was also measured at these time points. The sow's feed intake was calculated by the difference in feed allowance and feed refusals during the experimental period. After this, the average feed intake and body weight gain during the whole experimental period (day 109 of gestation until weaning) were calculated. The gestation length and number of piglets born (alive and still) were also recorded.

Piglets were weighed at birth and at weaning. The intake of creep feed was also recorded from day 14 up to day 26. Average feed intake and growth were calculated. Mortality rate was recorded in sows and piglets.

#### Levels of ZEN, DON, and Their Derivatives in Colostrum, Milk, and Serum Samples

Serum samples were collected from the sows before starting the feeding trial at day 109 of gestation. Colostrum samples were collected from the sows at farrowing for mycotoxin analysis. At weaning (day 26 after farrowing), milk and serum samples were collected from the sows and serum samples were collected from 10 piglets per sow. The method of analysis has previously been validated and described (15, 16). In brief, the analysis was performed on a 4,000 QTrap mass spectrometer equipped with an ESI source (Applied Biosystems, Darmstadt, Germany) and a 1,200 series LC system (Agilent Technologies, Böblingen, Germany). The analytical column was a Pursuit XRs Ultra C18 column (100 × 2 mm, 2.8 μm; Agilent Technologies). A binary gradient of LC-MS grade water as eluent A and MeOH–ACN (70:30) as eluent B was used to separate ZEN, DON, and their metabolites. The ESI-MS/MS was performed in negative mode using a multiple reaction monitoring (MRM) technique. The serum samples were prepared as described by (15). A slightly modified sample preparation method was used for milk and colostrum (16). LOD and LOQ values were calculated from low spiked milk and serum samples based on signal-to-noise ratios of 3:1 and 10:1 using the Analyst Software tool and the quantifier transition (16). Samples were not corrected for recovery, which was calculated as the ratio of the concentration obtained from the calibration curve and the known spiking level.

Transfer of ZEN and DON to colostrum and milk was calculated as previously described with a slight modification (17). In the present study, instead of calculating the carry-over factor, the percentage of transfer was calculated as mycotoxin concentration in the colostrum or milk divided by the mycotoxin exposure via feed. The toxin exposure was calculated by multiplying the toxin concentration of the diet with the feed intake of each sow and dividing it by the mean body weight (18) between the start of the trial and farrowing for the colostrum period, and between farrowing and weaning for the milk period.

#### Statistical Analysis

To determine the minimum number of replicates, a power analysis was performed based on a study from (19) where sows were fed diets contaminated with deoxynivalenol. This calculation was based on a probability of 5% and a power of 80%, with a SEM of 0.09 and a relevant difference of 0.64 kg of feed intake/day. The experimental data were analyzed with ANOVA (GenStat Version 20.0, 2020). Each sow was an experimental unit. Data collected from piglets (10 per sow) were used as a mean value per sow. Treatment means were compared by least significant difference (LSD). Values with *P* ≤ 0.05 were considered statistically significant. For all parameters, parity was used as a covariate to minimize the effect of age. Furthermore, the body weight and backfat thickness of the sow at the start of the experiment was used as a covariate for body weight and backfat development. The percentage of stillborn piglets, individual piglet weight at birth, and coefficient of variance at birth were corrected for the number of total born piglets. Pre-weaning litter growth and pre-weaning mortality were corrected for the number of piglets at standardization and weaning age.

## Results

### Sows and Litter Performance

The feed intake, body weight, and backfat development of the sows are shown in Table 3. Average individual feed intake during the whole experimental period (day 109 of gestation until weaning) was 4.9 kg/d. At the start of the experiment, sows had an average body weight of 269 kg. No effect of the dietary treatments was observed regarding sow's body weight gain or average daily feed intake (Table 3).

**Table 3:** Effect of dietary treatments on average daily feed intake (ADFI), body weight (BW), BW gain (BWG), backfat (BF), and BF gain (BFG)^*a*^.

	**T1** **(*n* = 5)**	**T2** **(*n* = 5)**	**T3** **(*n* = 5)**	**LSD**	***P*** **-value**
ADFI (kg/d)					
d109–weaning	5.1	4.8	4.9	1.30	0.87
Farrowing–weaning	5.5	5.4	5.6	1.40	0.95
**BW development (kg)**					
BW d109	267	269	269	42.6	>0.99
BW farrowing	253	247	249	8.88	0.38
BW weaning	220	218	224	26.4	0.87
BWG d109–farrowing	−15.4	−21.1	−19.5	8.88	0.38
BWG farrowing–weaning	−33.4	−29.3	−24.6	23.6	0.73
BWG d109–weaning	−48.8	−50.3	−44.1	26.4	0.87
**BF development (mm)**					
BF farrowing	15.2	14.7	14.7	1.08	0.55
BF weaning	12.7	13.3	12.2	1.90	0.48
BFG d109–farrowing	−0.14	−0.67	−0.59	1.08	0.55
BFG farrowing–weaning	−2.54	−1.36	−2.51	2.08	0.44
BFG d109–weaning	−2.67	−2.03	−3.10	1.90	0.48

a *All values for body weight and backfat thickness were corrected for body weight and backfat thickness of the sow at the start of the experiment; T1, LoZEN from d109 gestation until d26 of lactation; T2, HiZEN from d109 gestation until d26 of lactation; T3, HiZEN + algoclay-based mycotoxin decontaminant from d109 gestation until d26 of lactation. No effects of dietary treatment on litter size, litter weight, or individual piglet*.

No effects of dietary treatment on litter size, litter weight, or individual piglet weight were observed (Table 4). On average, sows in the experiment had 16.3 total born (TB). Litters were standardized between 24 and 48 h after birth at 12–14 piglets per litter, resulting in an average of 13.3 piglets per sow. For this, cross fostering was not necessary because of the average 14.9 live born (LB) piglets with an average birth weight of 1,355. Instead, some litters had more than 14 piglets. In this case, the extra LB piglets were transferred to sows outside the experiment.

**Table 4:** Effect of dietary treatment on performance of the sow at farrowing.

	**T1**	**T2**	**T3**	**LSD**	***P*** **-value**
Gestation length (d)	114.8	114.2	114.6	2.68	0.87
Number of piglets (*n*)					
Total born	14.8	17.0	17.0	4.89	0.55
Live born (LB)	14.0	16.0	14.6	4.04	0.56
Still born	0.8	1.0	2.4	2.11	0.24
Birth weight (LB)					
Litter (kg)	20.4	20.9	18.8	6.03	0.74
Individual (kg)^*^	1.4	1.3	1.3	0.39	0.95

a *Values are corrected for the number of total born piglets. T1, LoZEN from d109 gestation until d26 of lactation; T2, HiZEN from d109 gestation until d26 of lactation; T3, HiZEN + algoclay-based mycotoxin decontaminant from d109 gestation until d26 of lactation*.

Dietary treatments did not affect litter/piglet pre-weaning growth, nor did it affect pre-weaning mortality (Table 5). In this experiment, the piglets had an average weaning weight of 8.1 kg, which means an average daily gain of 245 g/d until weaning. No significant differences in weaning weight were observed among treatments.

**Table 5:** Effect of dietary treatment on piglet performance.

	**T1**	**T2**	**T3**	**LSD**	***P*** **-value**
Weaning age (d)	27.2	27.0	27.0	2.60	0.99
**Number of piglets**					
Start^*a*^	12.8	13.6	13.2	1.31	0.44
Weaning	11.6	12.8	12.2	1.98	0.45
**Weaning weight**					
Litter (kg)^*b*^	107.0	91.7	95.7	22.8	0.39
Piglet (kg)^*b*^	8.7	7.5	7.9	1.81	0.36
CV (%)^*b*^	12.3	18.3	18.2	9.66	0.35
**Growth**					
Piglet (g/d)^*b*^	267	225	242	0.61	0.35
Litter (kg/d)^*b*^	3.3	2.7	2.9	0.78	0.35
**Mortality**					
Pre-weaning^*a*^ (%)	6.5	8.4	7.9	10.16	0.92
**Creep feed intake**					
Piglet (g/d)	18.1	26.7	30.3	19.92	0.41

a *After standardization*.

b *Values are corrected for the number of piglets at standardization and weaning age. T1, LoZEN from d109 gestation until d26 of lactation; T2, HiZEN from d109 gestation until d26 of lactation; T3, HiZEN + algoclay-based mycotoxin decontaminant from d109 gestation until d26 of lactation. CV, Coefficient of variation*.

### Analysis of Biological Samples

#### Mycotoxins in Colostrum and Milk

A significant increase in α-ZEL levels was observed in the colostrum of sows from T2, i.e., those fed a HiZEN diet from d109 until weaning. These levels were even higher when the sows were fed the HiZEN diet supplemented with a decontaminant. However, no differences were observed among the treatments when evaluating the milk samples at weaning (Table 6). The percentage transfer of ZEN from feed to colostrum in the ZEN form was 0.004%, 0.002%, and 0.002% for T1, T2, and T3, respectively. The percentage transfer of ZEN from feed to milk in the ZEN form was 0.004%, 0.002%, and 0.002% for T1, T2, and T3, respectively. The percentage transfer of the sum ZEN and α-ZEL relative to ZEN intake was 0.011%, 0.008%, and 0.010% in the colostrum from T1, T2, and T3, respectively. For milk, the transfer of the sum ZEN and α-ZEL relative to ZEN intake was 0.006%, 0.003%, and 0.003% in the colostrum from T1, T2, and T3, respectively. Likewise, the percentage transfer of DON from feed to colostrum was low, being 0.03%, 0.03%, and 0.02% for T1, T2, and T3, respectively. The percentage transfer of DON from feed to milk was 0.04%, 0.05%, and 0.03% for T1, T2, and T3, respectively. These values did not differ significantly.

**Table 6:** Effect of dietary treatment on mycotoxin levels (ng/ml) in colostrum and milk.

	**T1**	**T2**	**T3**	**LSD**	***P*** **-value**
**Colostrum**					
ZEN	0.053	0.075	0.059	0.0535	0.67
α-ZEL	0.094^a^	0.218^b^	0.299^c^	0.0643	< 0.01
DON	0.75	0.76	0.71	1.183	> 0.99
**Milk**					
ZEN	0.120	0.115	0.110	0.0870	0.97
α-ZEL	0.062	0.156	0.121	0.1340	0.34
DON	2.98	2.37	1.61	1.893	0.32

a−c *Different superscripts indicate significant differences among treatments (P ≤ 0.05). T1, LoZEN from d109 gestation until d26 of lactation; T2, HiZEN from d109 gestation until d26 of lactation; T3, HiZEN + algoclay-based mycotoxin decontaminant from d109 gestation until d26 of lactation. β-ZEL, ZAN, α-ZAL, β-Zearalanol, and de-DON were not detected in the samples. DON, Deoxynivalenol; de-DON, De-epoxy-deoxynivalenol; ZEN, Zearalenone; α-ZEL, α-Zearalenol; β-ZEL, β-Zearalenol; ZAN, Zearalanone; α-ZAL, α-Zearalanol; β-ZAL, β-Zearalanol*.

#### Mycotoxins in Serum

At the start of the trial, no differences were observed in the serum levels of mycotoxins. However, at the end of the trial, i.e., day 26 of lactation, a significant increase was observed in the serum levels of ZEN, α-ZEL, and β-ZEL in sows fed diet T2 (HiZEN). Sows fed a HiZEN diet supplemented with a decontaminant (T3) showed a significant decrease in the serum levels of ZEN, α-ZEL, and β-ZEL, the latter to a level similar to the control (T1). Although there were no significant differences in the levels of ZEN and its derivatives in the serum of piglets, the serum levels of de-DON were significantly higher in the piglets from T2, but similar to the control in the piglets from T3 (Table 7).

**Table 7:** Effect of treatments on the mycotoxin levels (ng/ml) in the serum of sows and piglets.

	**T1**	**T2**	**T3**	**LSD**	***P*** **-value**
**Sows**					
Day 109 of gestation					
ZEN	0.066	0.066	0.050	0.0353	0.54
α-ZEL	0.148	0.258	0.155	0.1470	0.24
β-ZEL	0.057	0.081	0.063	0.0523	0.61
DON	0.445	0.929	0.445	0.5080	0.37
de-DON	-	-	-	-	-
Day 26 weaning					
ZEN	0.43^a^	1.11^c^	0.74^b^	0.295	<0.001
α-ZEL	0.94^a^	3.42^c^	2.40^b^	0.735	<0.001
β-ZEL	0.23^a^	0.53^b^	0.27^a^	0.111	<0.001
DON	3.07	2.10	3.26	1.817	0.36
de-DON	0.11	0.15	0.21	0.325	0.83
**Piglets at weaning**					
ZEN	0.024	0.024	0.017	0.0152	0.49
α-ZEL	0.011	0.040	0.015	0.0279	0.09
β-ZEL	0.042	0.041	0.040	0.0345	0.59
DON	0.045	0.099	0.062	0.0572	0.15
de-DON	0.040^a^	0.058^b^	0.040^a^	0.0160	0.05

a−c *Different superscripts indicate significant differences among treatments (P ≤ 0.05). T1: LoZEN from d109 gestation until d26 of lactation. T2: HiZEN from d109 gestation until d26 of lactation. T3: HiZEN + algoclay-based mycotoxin decontaminant from d109 gestation until d26 of lactation. ZAN, α-ZAL, β-ZAL were not detected in the samples. DON, Deoxynivalenol; de-DON, De-epoxy-deoxynivalenol; ZEN, Zearalenone; α-ZEL, α-Zearalenol; β-ZEL, β-Zearalenol; ZAN, Zearalanone; α-ZAL, α-Zearalanol; β-ZAL, β-Zearalanol*.

## Discussion

In the present study, we evaluated the ability of an algoclay-based mycotoxin decontaminant to limit the transmission of ZEN and DON to piglets during the last week of the sows' gestation and lactation. Exposure to ZEN and DON at the tested levels did not affect the performance of the sows and piglets. Importantly, the algoclay-based mycotoxin decontaminant did not impair the performance of the sows and piglets either. It is known that mycotoxin-adsorbing agents may interact with dietary nutrients (e.g., vitamins or minerals), impairing animal performance (20). Nevertheless, in 2006 it was demonstrated that the algoclay technology does not affect the bioavailability of nutrients (21).

Dietary exposure during the last week of gestation significantly increased the levels of α-ZEL in the colostrum of sows fed a diet containing 300 ppb ZEN when compared to a diet with 100 ppb ZEN. When the diet was supplemented with the test mycotoxin decontaminant, the levels of α-ZEL in the colostrum were even higher. However, when the milk was evaluated at weaning, no differences were observed among the dietary treatments. This may indicate a temporary effect of the mycotoxin decontaminant in enhancing the conversion of ZEN to α-ZEL, which probably occurred at the level of intestinal microbiota as an important site of ZEN metabolism (22) as the decontaminant is assumed to act in chyme through adsorption of mycotoxins. However, an additional interaction between the decontaminant and the physiological state of the sow probably contributed to the observed differences in the metabolization of ZEN between the colostrum and milk period. The nature of this interaction cannot be answered by the present experimental findings but might include differences in feed intake and consequently the mean retention time of ingesta, resulting in different periods of time for interactions between decontaminant, microbiota, ZEN and mucosal contact. Other explanations could include simple dilution/concentration effects mediated through varying colostrum/milk yields. However, there was no correlation between the number and weight of suckling piglets and the mycotoxin residues in colostrum and milk.

Serum levels of ZEN and α-ZEL of sows fed the HiZEN diet were much higher than those measured in the milk, i.e., 1.11 vs. 0.12 ng/ml and 3.42 vs. 0.16 ng/ml, respectively. The levels of DON in serum were similar to those detected in milk. The low levels of ZEN in milk were expected due to its low carry-over (23), which might be affected by the animal health status as previously shown in cattle (24). In the present trial, only healthy sows without reproductive disorders were selected for dietary exposure to the mycotoxin.

A significant increase in ZEN, α-ZEL, and β-ZEL levels was observed in the serum of sows fed the HiZEN diet, whereas dietary supplementation with the tested mycotoxin decontaminant significantly decreased the levels of ZEN and its metabolites in serum. In 2006, it was demonstrated that the intestinal absorption of mycotoxins (DON and fumonisins) was decreased in the presence of the algoclay technology used in this study (21). Thus, a reduction in intestinal absorption in the presence of the algoclay-based mycotoxin decontaminant is likely to be responsible for the decrease in the levels of ZEN and its metabolites in serum.

When the HiZEN diet was supplemented with the algoclay-based mycotoxin decontaminant, the de-DON levels were similar to those of control piglets. Furthermore, there was a trend toward increased α-ZEL levels in the serum of piglets from the HiZEN diets, and this effect was counteracted by the test algoclay-based mycotoxin decontaminant. In a previously published study (6) we demonstrated that piglets from the HiZEN-exposed sows had an increased serum level of α-ZEL together with a decrease in serum oestradiol levels, showing that this low exposure was sufficient to influence the oestrogenic activity in piglets. Although the α-ZEL level in the serum of piglets was only numerically decreased when the algoclay-based mycotoxin decontaminant was present in the HiZEN diet, the levels of this mycotoxin were significantly decreased in the serum of sows, indicating that the exposure of piglets should also be minimized.

## Conclusions

In a previous study, it was demonstrated that dietary exposure of sows to 300 ppb ZEN will result in increased milk transmission of this mycotoxins and metabolites to piglets, which will experience an inflammatory process (6). In the present study, the tested algoclay-based mycotoxin decontaminant was able to decrease the levels of ZEN and its metabolites in the serum of sows and decrease de-DON in the serum of piglets. The temporal increase in α-ZEL in the colostrum of decontaminant-supplemented sows requires further clarification. Although no effects on performance were observed and the transfer of ZEN from feed to milk is low (0.002%), it is important to note that piglets often experience stress immediately after weaning. Therefore, methods to minimize the exposure to mycotoxins during lactation may avoid secondary diseases or other factors leading to impaired performance of piglets during growth. The present test algoclay-based mycotoxin decontaminant did not affect performance. Whether the decontaminant-mediated reduction in the bioavailability of ZEN and DON has long-term effects on the reproductive performance of both the sows and their offspring needs to be clarified further.

## Data Availability Statement

The original contributions presented in the study are included in the article/Supplementary Material, further inquiries can be directed to the corresponding authors.

## Ethics Statement

This study was conducted according to the guidelines of the Animal and Human Welfare Codes/Laboratory practice codes in the Netherlands. The protocol was approved by the Ethics Review Committee: Body of Animal Welfare at SFR (AVD246002015280), approval date: 8 January 2019.

## Author Contributions

FM and RS conceived and designed this study. XB and RS performed the experiments. XB, RS, and JS conducted the analysis. FM, JL, and MR acquired the funding. FM, SD, and RS supervised the study. XB, JS, and RS wrote the original draft. XB, JS, JL, MR, FM, SD, and RS revised and edited the final version of the manuscript. All authors have read and agreed to the published version of the manuscript.

## Conflict of Interest

The authors declare that the research was conducted in the absence of any commercial or financial relationships that could be construed as a potential conflict of interest.

## Publisher's Note

All claims expressed in this article are solely those of the authors and do not necessarily represent those of their affiliated organizations, or those of the publisher, the editors and the reviewers. Any product that may be evaluated in this article, or claim that may be made by its manufacturer, is not guaranteed or endorsed by the publisher.

## References

[1] EFSA CONTAM Panel (EFSA Panel on Contaminants in the Food Chain) Knutsen HK Alexander J Barregard L Bignami M Bruschweiler B. Scientific Opinion on the risks to human and animal health related to the presence of deoxynivalenol and its acetylated and modified forms in food and feed. EFSA J. (2017) 15:4718. 10.2903/j.efsa.2017.4718 32625635 PMC7010102

[2] EFSA CONTAM Panel (EFSA Panel on Contaminants in the Food Chain) Knutsen HK Alexander J Barregard L Bignami M Bruschweiler B. Scientific opinion on the risks for animal health related to the presence of zearalenone and its modified forms in feed. EFSA J. (2017) 15:4851. 10.2903/j.efsa.2017.4851 32625539 PMC7009830

[3] Schoevers EJ Santos RR Colenbrander B Fink-Gremmels J Roelen BAJ. Transgenerational toxicity of zearalenone in pigs. Reprod Toxicol. (2012) 34:110–9. 10.1016/j.reprotox.2012.03.004 22484360

[4] Stastny K Stepanova H Hlavova K Faldyna M. Identification and determination of deoxynivalenol (DON) and deepoxydeoxynivalenol (DOM-1) in pig colostrum and serum using liquid chromatography in combination with high resolution mass spectrometry (LC-MS/MS (HR)). J Chromatogr B Analyt Technol Biomed Life Sci. (2019) 1126–27:121735. 10.1016/j.jchromb.2019.121735 31394401

[5] Trevisi P Luise D Spinelli E Correa F De Leo E Trambajolo G. Transfer of mycotoxins from lactation feed to colostrum of sows. Animals. (2020) 10:e2253. 10.3390/ani10122253 33266144 PMC7761246

[6] de Grave XB Saltzmann J Laurain J Rodriguez MA Molist F Dänicke S. Transmission of zearalenone, deoxynivalenol, and their derivatives from sows to piglets during lactation. Toxins. (2021) 6:e37. 10.3390/toxins13010037 33419041 PMC7825292

[7] European Union Register Of Feed Additives Pursuant To Regulation (EC) no 1831/2003 Appendixes 3e & 4(I). Annex I: List of additives. Available online at: http://ec.europa.eu/food/food/animalnutrition/feedadditives/comm_register_feed_additives_1831-03.pdf (accessed 4 Jan, 2015).

[8] Burt R. Soil Survey Investigations Report No 45. Lincoln, NE, USA: USDA-NRCS (2011).

[9] De Mil T Devreese M De Baere S Van Ranst E Eeckhout M De Backer P. Characterization of 27 mycotoxin binders and the relation with in vitro zearalenone adsorption at a single concentration. Toxins. (2015) 7:21–33. 10.3390/toxins7010021 25568976 PMC4303810

[10] Jaynes WF Zartman RE Hudnall WH. Aflatoxin B1 adsorption by clays from water and corn meal. Appl Clay Sci. (2007) 36:197–205. 10.1016/j.clay.2006.06.012

[11] Laza AL. Développement d'une nouvelle alternative naturelle en alimentation et hygiène animale à base d'argile et d'algues. Doctoral thesis, de Haute Alsace, Mulhouse, France (2006).

[12] Balusson H Laza-Knoerr AL Balusson S Blouin S Vintan D. Method for Preparing An Intercalated And/Or Exfoliated Organophillic Clay From Clay and Macroalgae, Corresponding Fertiliser Product, Food Supplement for Animals and Fish Feed. Patent number EP2674397, PARIS: European Patent Office Bulletin 2013/51 (2013).

[13] Lahaye M Robic A. Structure and functional properties of Ulvan, a polysaccharide from green seaweeds. Biomacromolecules. (2007) 8:1765–74. 10.1021/bm061185q 17458931

[14] The European Commission. Commission recommendation 2016/1319/EC of 29 July 2016 amending commission recommendation 2006/576/EC on the presence of deoxynivalenol, zearalenone, ochratoxin A, T-2 and HT-2 and fumonisins in products intended for animal feeding. Off J Eur Union. (2016) L208:58–60.

[15] Brezina U Valenta H Rempe I Kersten S Humpf H-U Dänicke S. Development of a liquid chromatography tandem mass spectrometry method for the simultaneous determination of zearalenone, deoxynivalenol and their metabolites in pig serum. Mycotoxin Res. (2014) 30:171–86. 10.1007/s12550-014-0200-8 24925826

[16] Winkler J Kersten S Valenta H Meyer U Engelhardt UH Dänicke S. Development of a multi-toxin method for investigating the carryover of zearalenone, deoxynivalenol and their metabolites into milk of dairy cows. Food Addit Contam Part A Chem Anal Control Expo Risk Assess. (2015) 32:371–80. 10.1080/19440049.2015.1011714 25849036

[17] Goyarts T Dänicke S Valenta H Ueberschär KH. Carry-over of *fusarium* toxins (deoxynivalenol and zearalenone) from naturally contaminated wheat to pigs. Food Addit Contam. (2007) 24:369–80. 10.1080/02652030600988038 17454110

[18] Winkler J Kersten S Meyer U Engelhardt U Dänicke S. Residues of zearalenone (ZEN), deoxynivalenol (DON) and their metabolites in plasma of dairy cows fed fusarium contaminated maize and their relationships to performance parameters. Food Chem Toxicol. (2014) 65:196–204. 10.1016/j.fct.2013.12.020 24361404

[19] Sayyari A Framstad T Krodenaes AK Sivertsen T. Effects of feeding naturally contaminated deoxynivalenol diets to sows during late gestation and lactation in a high-yield specific pathogen-free herd. Porcine Health Manag. (2018) 4:26. 10.1186/s40813-018-0102-9 30410782 PMC6211461

[20] Boudergue C Burel C Dragacci S Favrot MC Fremy JM Massimi C. Review of mycotoxin-detoxifying agents used as feed additives: mode of action, efficacy and feed/food safety. EFSA J. (2009) 6:192. 10.2903/sp.efsa.2009.EN-22

[21] Havenaar R Demais H. Efficacy of sequestrant/chelator amadeite in the binding of mycotoxins during transit through a dynamic gastrointestinal model (TIM) simulating the GI conditions of pigs. In: Proceedings of the 4th World Mycotoxin Forum. Cincinnati, OH (2006).

[22] Dänicke S Winkler J. Invited review: diagnosis of zearalenone (ZEN) exposure of farm animals and transfer of its residues into edible tissues (carry over). Food Chem Toxicol. (2015) 84:225–49. 10.1016/j.fct.2015.08.009 26277628

[23] Liu J Applegate T. Zearalenone (ZEN) in livestock and poultry: dose, toxicokinetics, toxicity and estrogenicity. Toxins. (2020) 12:377. 10.3390/toxins12060377 32517357 PMC7354539

[24] Fink-Gremmels J. Mycotoxins in cattle feeds and carry-over to dairy milk: a review. Food Addit Contam: Part A Chem Anal Control Expo Risk Assess. (2008) 25:172–80. 10.1080/02652030701823142 18286407

