# Supplementary material for: The Ability of an Algoclay-Based Mycotoxin Decontaminant to Decrease the Serum Levels of Zearalenone and Its Metabolites in Lactating Sows
Source: Front Vet Sci. 2021 Sep 30;8:704796. doi: 10.3389/fvets.2021.704796 (PMC8515040; doi:10.3389/fvets.2021.704796)
Supplement: Supplementary file 1 [file Data_Sheet_1.DOCX]

Table S1. Composition (%) and calculated nutrient levels (g/kg) of the sows gestation diet

| **INGREDIENTS** | | |  |
| --- | --- | --- | --- |
|  |  | Barley | 23.5 |
|  |  | Wheat middlings | 21.6 |
|  |  | Soybean hulls 32-36%CF | 9.3 |
|  |  | Palm kernel meal <20% CF | 8.0 |
|  |  | Wheat | 7.5 |
|  |  | Rye | 6.7 |
|  |  | Sugar beet Pulp <10% Sugar | 5.3 |
|  |  | Maize | 5.0 |
|  |  | Rapeseed meal 0=0 | 4.8 |
|  |  | Molass. Beet | 2.0 |
|  |  | Palm oil | 1.2 |
|  |  | Sunflower seed meal 38% CP | 1.0 |
|  |  | Lecithine mix | 1.0 |
|  |  | Limestone | 0.82 |
|  |  | Sodiumbicarbonate | 0.64 |
|  |  | Prem Sow (incl Fytase) | 0.63 |
|  |  | Organic acid mix | 0.6 |
|  |  | Monocalciumphosphate | 0.50 |
|  |  | Salmon oil | 0.3 |
|  |  | Lysine-HCl (L 79%) | 0.12 |
|  |  | Threonine (L 98%) | 0.03 |
| **NUTRIENTS** | |  |  |
| g/Kg |  | Moisture | 115 |
| g/Kg |  | Ash | 49 |
| g/Kg |  | Crude Protein | 126 |
| g/Kg |  | Crude Fibre | 102 |
| g/Kg |  | Sugar | 48 |
| g/Kg |  | Starch_EW | 289 |
| g/Kg |  | CFat_AH | 51 |
| Mj/kg |  | NE | 8.6 |
| g/Kg |  | SID_LYSs | 4.8 |
| g/Kg |  | SID_METs | 1.66 |
| g/Kg |  | SID_M+Cs | 3.6 |
| g/Kg |  | SID_THRs | 3.4 |
| g/Kg |  | SID_TRPs | 1.1 |
| g/Kg |  | SID_VALs | 4.6 |
| g/Kg |  | SID_LEUs | 6.5 |
|  |  | SID met/SID lys | 0.35 |
|  |  | SID m+c/SID lys | 0.75 |
|  |  | SID threo/SID lys | 0.72 |
|  |  | SID val/SID lys | 0.96 |
|  |  | SID tryp/SID lys | 0.24 |
|  |  | SID leu/SID lys | 1.37 |
| g/Kg |  | Ca | 5.9 |
| g/Kg |  | Na | 2.2 |
| g/Kg |  | Cl | 2.1 |
| g/Kg |  | K | 8.7 |
| g/Kg |  | avCas | 6.7 |
| meq |  | dEB | 282 |
| g/Kg |  | ATTD-P | 2.9 |
| g/Kg |  | FCHO | 207 |
| g/Kg |  | ICHO | 123 |

Table S2. Composition (%) and calculated nutrient levels (g/kg) of the sows experimental diet

| **INGREDIENTS** | | |  |
| --- | --- | --- | --- |
|  |  | Corn | 40.0 |
|  |  | Wheat | 12.1 |
|  |  | Sunflower seed meal | 10.0 |
|  |  | Wheat middling’s | 7.24 |
|  |  | Soybean hulls | 7.50 |
|  |  | Sugar beet pulp | 5.00 |
|  |  | Molasses Cane | 4.00 |
|  |  | Linseed | 3.68 |
|  |  | Soybean meal | 3.01 |
|  |  | Potato | 2.23 |
|  |  | Palm oil | 0.50 |
|  |  | Soybean oil | 0.50 |
|  |  | Limestone | 1.25 |
|  |  | Sodium bicarbonate | 0.35 |
|  |  | Monocalcium phosphate | 0.86 |
|  |  | Salt | 0.21 |
|  |  | Lysine-HCL (L 79%) | 0.32 |
|  |  | Threonine (L 98%) | 0.24 |
|  |  | Methionine (DL 99%) | 0.00 |
|  |  | Tryptophan (L 98%) | 0.01 |
|  |  | Phytase Sow | 0.10 |
|  |  | Premix Gestation | 0.50 |
|  |  | Premix Lactation | 0.40 |
| **NUTRIENTS** | |  |  |
| g/Kg |  | Moisture | 122.1 |
| g/Kg |  | Crude Protein | 150.0 |
| g/Kg |  | Ash | 58.8 |
| g/Kg |  | Crude Fibre | 70.4 |
| g/Kg |  | Sugar | 51.9 |
| g/Kg |  | Crude Fat | 46.6 |
| g/Kg |  | Starch | 334.1 |
| MJ/kg |  | NE | 2269 |
| g/Kg |  | SID_LYSs | 7.6 |
| g/Kg |  | SID_METs | 2.4 |
| g/Kg |  | SID_M+Cs | 4.4 |
| g/Kg |  | Ca | 9.3 |
| g/Kg |  | P | 5.7 |
| g/Kg |  | Na | 2.1 |
| g/Kg |  | Cl | 3.3 |
| g/Kg |  | K | 8.4 |
| meq |  | dEB | 213 |
| mg/kg |  | Cu | 23.1 |
| mg/kg |  | Zn | 139.6 |
|  |  | US ratio | 4.6 |
| g/Kg |  | FCHO | 160.0 |
| g/Kg |  | iCHO | 80.0 |
| g/Kg |  | NSPs | 226.4 |

Table S3. Composition (%) and calculated nutrient levels (g/kg) of the creep feed

| **INGREDIENTS** |  | **Creep feed** |
| --- | --- | --- |
|  | Potato protein | 1.30 |
|  | Barley | 16.00 |
|  | Oat flakes | 10.00 |
|  | Coconut oil | 1.50 |
|  | Milk powder skimmed | 2.50 |
|  | Soybean oil | 1.75 |
|  | Sugar Feed grade | 3.20 |
|  | Wheat | 18.62 |
|  | Wheat middling’s | 6.20 |
|  | Monocalcium phosphate | 1.26 |
|  | P start 2220 0,4% | 0.40 |
|  | Whey powder sweet | 10.00 |
|  | Salt | 0.33 |
|  | Prem. vitamin ad3e | 0.10 |
|  | Premix Copper-pigs | 1.74 |
|  | Maize expanded | 13.33 |
|  | Soycomil (spc) | 4.20 |
|  | Vitamins/trace-elem. | 0.10 |
|  | Lysine-HCL (L 79%) | 0.50 |
|  | Methionine (DL 99%) | 0.14 |
|  | Threonine (L 98%) | 0.16 |
|  | Tryptophane (L 98%) | 0.05 |
|  | Wheat gluten meal | 1.40 |
|  | Valine (L 99%) | 0.03 |
|  | Sodium bicarbonate | 0.10 |
|  | Fish oil | 0.50 |
|  | Limestone | 0.70 |
|  | Soya bean meal | 3.90 |
| **NUTRIENTS** |  |  |
| g/Kg | Moisture | 102.29 |
| g/Kg | Crude protein | 167.10 |
| g/Kg | Ash | 53.95 |
| g/Kg | Crude fibre | 23.59 |
| g/Kg | Sugar | 135.82 |
| g/Kg | Fat | 65.42 |
| g/Kg | Starch | 342.70 |
| MJ/kg | NE | 10.21 |
| g/Kg | SID_LYSs | 11.07 |
|  | SID met/SID lys | 0.35 |
|  | SID m+c/SID lys | 0.58 |
